# Purinergic Signaling Induces Cyclooxygenase-1-Dependent Prostanoid Synthesis in Microglia: Roles in the Outcome of Excitotoxic Brain Injury

**DOI:** 10.1371/journal.pone.0025916

**Published:** 2011-10-14

**Authors:** Josef Anrather, Eduardo F. Gallo, Takayuki Kawano, Marcello Orio, Takato Abe, Camile Gooden, Ping Zhou, Costantino Iadecola

**Affiliations:** Division of Neurobiology, Department of Neurology and Neuroscience, Weill Cornell Medical College, New York, New York, United States of America; Universidad Federal de Santa Catarina, Brazil

## Abstract

Cyclooxygenases (COX) are prostanoid synthesizing enzymes constitutively expressed in the brain that contribute to excitotoxic neuronal cell death. While the neurotoxic role of COX-2 is well established and has been linked to prostaglandin E_2_ synthesis, the role of COX-1 is not clearly understood. In a model of *N*-Methyl-D-aspartic acid (NMDA) induced excitotoxicity in the mouse cerebral cortex we found a distinctive temporal profile of COX-1 and COX-2 activation where COX-1, located in microglia, is responsible for the early phase of prostaglandin E_2_ synthesis (10 minutes after NMDA), while both COX-1 and COX-2 contribute to the second phase (3–24 hours after NMDA). Microglial COX-1 is strongly activated by ATP but not excitatory neurotransmitters or the Toll-like receptor 4 ligand bacterial lipopolysaccharide. ATP induced microglial COX-1 dependent prostaglandin E_2_ synthesis is dependent on P2X7 receptors, extracellular Ca^2+^ and cytoplasmic phospholipase A2. NMDA receptor activation induces ATP release from cultured neurons leading to microglial P2X7 receptor activation and COX-1 dependent prostaglandin E_2_ synthesis in mixed microglial-neuronal cultures. Pharmacological inhibition of COX-1 has no effect on the cortical lesion produced by NMDA, but counteracts the neuroprotection exerted by inhibition of COX-2 or observed in mice lacking the prostaglandin E_2_ receptor type 1. Similarly, the neuroprotection exerted by the prostaglandin E_2_ receptor type 2 agonist butaprost is not observed after COX-1 inhibition. P2X7 receptors contribute to NMDA induced prostaglandin E_2_ production *in vivo* and blockage of P2X7 receptors reverses the neuroprotection offered by COX-2 inhibition. These findings suggest that purinergic signaling in microglia triggered by neuronal ATP modulates excitotoxic cortical lesion by regulating COX-1 dependent prostanoid production and unveil a previously unrecognized protective role of microglial COX-1 in excitotoxic brain injury.

## Introduction

Cyclooxygenases, enzymes that synthesize prostanoids (i.e. prostaglandins, thromboxanes and prostacyclines) from arachidonic acid, have been implicated in the mechanisms of a wide variety of pathological conditions, including several brain diseases [1]. Two genetically distinct isoforms of COX have been identified: COX-1 and COX-2. While COX-1 contributes to the production of prostanoids involved in normal cellular functions and enzyme levels are generally stable in cells that express this isozyme, COX-2 is highly inducible at the transcriptional level and is upregulated in different organs in pathological states associated with inflammation [2], [3].

In brain, COX-2 is constitutively expressed in neurons [4] and has been implicated in the development of brain lesions in models of excitotoxicity, hypoxia-ischemia, trauma, neurodegeneration and neuroinflammation [5]. The neurotoxic effect of COX-2 is mediated by prostaglandin (PG) E_2_ via activation of the PGE_2_ receptor subtype 1 (EP1) on neurons [6], [7]. PGE_2_ can also induce neuroprotection by activating neuronal EP2 receptors [8]. However, in models of inflammation or Aß neurotoxicity microglial EP2 receptor activation is deleterious, an effect mediated by the release of cytotoxic factors from microglia [9]. Thus, PGE_2_ may have beneficial or deleterious effects depending on its cellular source, the type of EP receptor expressed in target cells, and the cell type expressing EP receptors.

The role of COX-1 in neurotoxicity is far less clear. Although it has been suggested that both COX-1 and COX-2 contribute to NMDA-induced PGE_2_ production and free radical damage in hippocampus [10], COX-1 is not involved in excitotoxic cell death in a model of NMDA neurotoxicity in the cortex [11]. Similarly, both COX-1 and COX-2 have been reported to contribute to hippocampal damage after global cerebral ischemia [12], but COX-1 is protective in a model of focal cerebral ischemia [11]. These conflicting observations indicate that the role of COX-1 is complex and may depend on the cell type expressing COX-1 and on the context in which the injury is produced.

Therefore, in the present study we addressed the relative contribution of COX-1 and COX-2 to the PGE_2_ production and brain damage produced by cortical injection of NMDA. We found that COX-1, expressed mainly in microglia, is a major source of PGE_2_ in the early stages of NMDA neurotoxicity. Although COX-1 does not directly contribute to the injury, COX-1 enzymatic activity is required for the protective effect afforded by COX-2 inhibition, EP1 receptor deletion or EP2 receptor activation. Furthermore we find that microglial COX-1 is activated through purinergic signaling initiated by neuronal ATP release, leading to activation of P2X7 receptors and Ca^2+^-dependent phospholipase A2. The findings unveil a previously unrecognized neuroprotective role of purinergic signaling and microglial COX-1 in excitotoxic brain injury.

## Methods

### Reagents

NMDA, MK-801, ATP, periodate-oxidized ATP (oxATP), 2′(3′)-O-(4-Benzoylbenzoyl)-ATP (BzATP), and bacterial lipopolysaccharide (LPS) were from Sigma (St. Louis, MO). NS398, SC560, Butaprost, and Bromoenol lactone (BEL) were from Cayman Chemicals, Ann Arbor, MI). Arachidonyl trifluoromethyl ketone (AACOCF3) was from Biomol (Plymouth Meeting, PA). A438079 was from Tocris (Ellisville, MO). Neurobasal medium and B27 supplement were from Invitrogen (Carlsbad, CA). Fetal bovine serum and Penicillin/Streptomycin solution were from Atlanta Biologicals (Lawrenceville, GA). All other cell culture reagents were from MediaTech Inc. (Herndon, VA). All other chemicals were purchased from Sigma.

### Animals, surgical procedures, and drug treatments

All experimental procedures were approved by the Institutional Animal Care and Use Committee of Weill Cornell Medical College (Approval Number: 0710-678A). C57BL/6J mice were obtained from Jackson Laboratories (Bar Harbor, ME). EP1^−/−^, nNOS^−/−^, and P2X7^−/−^ mice were supplied from in house colonies [7], [13], [14], [15]. Null mice were on a C57Bl6 background and C57Bl6 mice were used as wild type controls. Male mice (age 2–3 months) were used for all experiments.

#### 1. Determination of cortical lesion volumes after local NMDA injections

As described in detail elsewhere [16], cortical NMDA injections (20 nmol in 200 nl) were carried out in isoflurane anesthetized mice (2% in oxygen) using a micropipette stereotaxically placed in the parietal cortex at 1.5 mm posterior to the bregma, 4.0 mm lateral and 0.8 mm below the dura. Lesion volumes were determined after 24 hours in Nissl-stained coronal brain sections using a computerized image analysis system [16], [17]. Where indicated, animals received NS398 (20 mg/kg; i.p.) or vehicle (200 µl 0.1 M Na_2_HPO_4_ pH 7.0; i.p.) 1 hour before and 1 hour after cortical NMDA injections. SC560 (5 mg/kg; i.p.) or vehicle (100 µl 10%DMSO in physiol. Saline; i.p.) was given 1 hour before and 6 hours after cortical NMDA injections. Doses and administration schedule were chosen based on the results of previous studies [16], [18]. The selectivity and specificity of SC560 and NS398 at the doses used was demonstrated previously [17], [19]. Some animals were injected with 20 nmol NMDA together with 170 fmol Butaprost or vehicle (0.014% ethanol). This dose of Butaprost has been shown to be neuroprotective in this model [16]. In some animals oxATP (300 nmol in 3 µl physiol. saline) or vehicle (3 µl physiol. saline) was stereotaxically delivered to the right lateral ventricle (0.5 mm posterior to the bregma, 1.0 mm lateral, 2.0 mm below the dura) 30 minutes before NMDA injections. The dosage was chosen to reflect the effective concentration of oxATP used for *in vitro* experiments (1 mM). The specific competitive P2X7 receptor antagonist A438079 (200 µmol/kg in physiol. saline; i.p.) or vehicle (100 µl physiol. saline; i.p.) was given 1 hour before cortical NMDA injections. This dosage has been shown to induce anti-nociceptive behavior in a model of neurophatic pain in mice [20].

#### 2. Measurment of cortical PGE_2_ levels after local NMDA injections

Animals were treated with SC560 and/or NS398 or respective vehicles as described above. Cortical NMDA injections were carried out as described above. Some animals were i.c.v. injected with oxATP or corresponding vehicle as described above. Where indicated, the non-competitive NMDA receptor antagonist MK-801 (3 mg/kg in physiol. saline; i.p.) or vehicle (175 µl physiol. saline; i.p.) was administered 30 minutes before cortical NMDA injections. At indicated time points animals were deeply anesthetized with isoflurane, decapitated and brains removed. The cortex at the site of injection (10–12 mg wet weight) was dissected and frozen in liquid nitrogen. Care was taken to limit the time for tissue harvesting to 45–55 seconds. Tissue extracts were prepared, purified over C18 cartridge columns and ELISA was performed according to manufacturer's recommendations (Cayman Chemicals).

### Immunofluorescence staining and confocal microscopy

Brains were removed from animals transcardially perfused with 4% paraformaldehyde and post-fixed overnight in 4% paraformaldehyde containing 20% sucrose. Coronal sections were collected on a cryostat. Injection needle tracks were visualized in Nissl stained sections and adjacent sections were used for immunofluorescence staining. The location of the cortical lesion eight hours after NMDA injection was determined by the clearly visible opacity of the lesioned area. Frozen sections were blocked with serum and incubated with respective primary antibodies directed against neuronal nuclear antigen (NeuN, clone A60, mouse IgG1, Chemicon, Temecula, CA, 1∶1000), glial fibrillary acidic protein (GFAP, clone GA5, mouse IgG1, Sigma, 1∶5000), ionized calcium binding adaptor molecule 1 (Iba1, rabbit polyconal, Wako, 1∶500), COX-1 (goat polyclonal, sc-1754, Santa Cruz Biotechnology, Santa Cruz, CA, 1∶200), or COX-2 (goat polyclonal, sc-1746, Santa Cruz Biotechnology, 1∶100). Respective secondary antibodies were incubated for 1 hour at room temperature (donkey-anti-mouse IgG-FITC, donkey-anti-rabbit IgG-FITC, donkey-anti-goat-Cy5; all at 1∶200, Jackson Immunoresearch, West Grove, PA). Mounted sections were analyzed on a SP5 confocal laser scanning microscope (Leica Microsystems, Bannockburn, IL) using adequate excitation lines and emission bands. No spectral overlap was observed between FITC and Cy5 acquisition channels. Data are presented as average orthographic projections of 40–48 images obtained at 200 nm Z-axis resolution.

### Cell cultures

Neuronal cultures were established from cortices of E17 C57/Bl6J mouse embryos as described [7] and used between days 11–13 in culture. We have previously shown that COX-2 is expressed in neurons cultured under these conditions [7]. Purity of neuronal cultures was estimated to be above 95% by immunofluorescent cell staining using antibodies directed against NeuN and GFAP. Mixed glia were cultured from neonatal C57/Bl6J mice and microglia were prepared after 21 days in culture by limited trypsinization as described elsewhere [21]. Microglia were used for experiments 2–3 days after trypsinization. In some experiments COX1^−/−^ (Taconic; [22]) or P2X7^−/−^ microglial cultures were used. Purity of preparations was assessed by indirect immunofluorescence staining using anti-F4/80 (microglial marker) and anti-GFAP antibodies or by flow cytometry using a CD11b specific antibody. Microglia constituted >97% of total cells. Neuron-microglia co-cultures were obtained by seeding 5×10^4^ microglia isolated from mixed glial cultures by shaking the cultures at 37°C [23] on top of neurons cultured in 12well plates as described above. Co-cultures were used within 1 hour after seeding. Independent experiments were repeated on cells derived from different isolations.

### PGE_2_ measurements in neuronal and glial cultures

PGE_2_ released from cultured cells was measured in cell culture supernatants. Cells were pre-treated with vehicle (DMSO at 0.033% final concentration) or inhibitors added directly to the growth medium for 1 hour. Cells were then washed twice in warm physiologic salt solution (PSS: NaCl 130, CaCl_2_ 1.8, Glucose 20, HEPES 10, and KHCO_3_ 3 mM, pH 7.35) and incubated in PSS containing inhibitors and/or agonists for 30 minutes after which cell culture supernatants were collected. LPS treatments lasting longer than 30 minutes were carried out in growth medium. 30 minutes before end of stimulation cells were washed twice and incubated in PSS as described above. PGE_2_ ELISA was performed according to manufacturer's recommendations (Cayman Chemicals).

### Determination of microglial cyclooxygenases expression

Cultured microglia were stimulated with LPS (1 µg/ml) for indicated times, washed in PBS and lysed in Laemmli buffer. Western blotting was carried out essentially as described [24] by probing membranes with COX-1 or COX-2 specific antibodies. Equal loading was assured by probing membranes for β-actin.

### Extracellular ATP measurements in neuronal cultures

Neurons were exposed to NMDA (1 mM) added directly to the culture medium. Supernatant was carefully removed to avoid cell aspiration and centrifuged (5000 g, 3 minutes at 4°C) to eliminate any cellular components. ATP in the medium was measured in duplicate samples by the luciferin/luciferase reaction in a plate luminometer (Berthold, Oak Ridge, TN) as suggested by the manufacturer (Promega, Madison, WI). All samples were processed within 30 minutes after harvest.

### Data analysis

Data are expressed as means ± SEM. Two-group comparisons were analyzed by the two-tailed t test for dependent or independent samples, as appropriate. Multiple comparisons were evaluated by ANOVA and Tukey's tests. Statistical significance was considered for p<0.05.

## Results

### Time-course of PGE_2_ production after cortical NMDA injection

**Figure 1:**
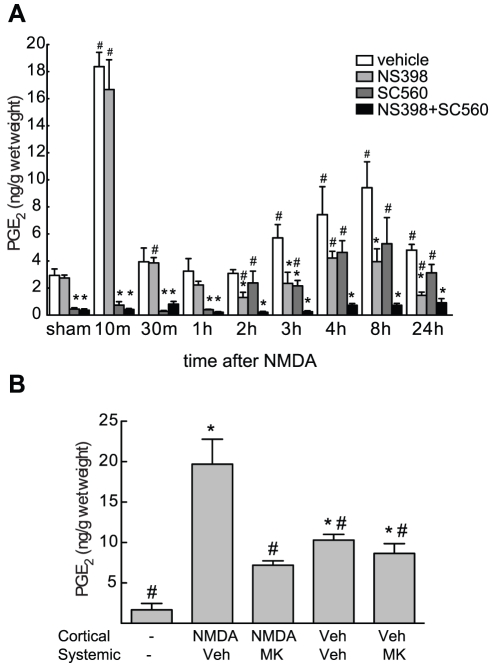
COX-1 and COX-2 contribute to PGE_2_ production after cortical NMDA injections. (**A**) Time course of PGE_2_ production after cortical NMDA injections. NMDA (20 nmol) was injected stereotactically into the parietal cortex. Were indicated animals received NS398 and/or SC560 as described in *Methods*. Vehicle consisted of 100 µl DMSO∶Saline (1∶9) administered i.p. PGE_2_ tissue levels were measured by ELISA (n = 5/group, * p<0.05 from vehicle treated group at the same time point, # p<0.05 from sham receiving the same systemic drug treatment). (**B**) Animals were treated with MK-801 (MK, 3 mg/kg; i.p.) or vehicle (175 µl 0.9% saline; i.p.) 30 minutes before cortical injections of NMDA (20 nmol in 200 nl) or vehicle (200 nl of 0.1 M phosphate buffer, pH 7.4). Tissue was collected 10 minutes after cortical injection. PGE_2_ tissue levels were measured by ELISA (n = 4/group, * p<0.05 from sham, # p<0.05 from NMDA+Sal).

First, we investigated the time course of PGE_2_ production in mice after cortical NMDA injections. Basal levels of PGE_2_ (2.9±0.5 ng/g tissue; n = 5) were comparable to those previously described [7]. NMDA injection elicited a biphasic increase in PGE_2_, characterized by a rapid increase at 10 min, followed by a return to baseline between 30 min and 2 hrs, and a slower increase at 8 hrs (Fig. 1A). The rapid PGE_2_ reduction between 10 and 30 min after NMDA is consistent with the rate of clearance of prostanoids in the mouse cortex [25]. Basal levels of PGE_2_ were markedly attenuated in mice treated with the COX-1 inhibitor SC560 (−85±3% compared to vehicle treated animals), while the COX-2 inhibitor NS398 had no effect on basal PGE_2_ (Fig. 1A). The early peak of PGE_2_ production was virtually abolished by SC560 (−96±2% compared to vehicle), but was not affected by NS398 (Fig. 1A). In contrast, the late increase in PGE_2_ was attenuated by both SC560 and NS398, although NS398 was more effective at 8 (−58±10% compared to vehicle) and 24 hours (−70±5% compared to vehicle) (Fig. 1A). Co-administration of both COX-1 and COX-2 inhibitors blocked NMDA-induced PGE_2_ production completely at all time points (p<0.05 from vehicle) (Fig. 1A). To rule out the possibility that the PGE_2_ production seen at the early time point was caused by the trauma of the micropipette insertion and not by NMDA receptor activation, we performed the vehicle and NMDA injections with or without the NMDA receptor antagonist MK-801. Treatment with MK-801 markedly attenuated PGE_2_ levels 10 min after NMDA injection (Fig. 1B). Vehicle injection caused a significant increase in PGE_2_ that was however not suppressed by MK-801, but was significantly lower than that observed after NMDA injection (Fig. 1B). This data suggest that trauma contributes moderately to the early PGE_2_ increase after cortical NMDA injections by a NMDA receptor-independent mechanism. In summary, COX-1 contributes to basal PGE_2_ production and to the early PGE_2_ peak after cortical NMDA injections, while both enzymes contribute to PGE_2_ synthesis at later time points.

### COX-1 inhibition reverses the protection exerted by COX-2 inhibition, EP1 receptor deletion, or EP2 receptor activation, but not nNOS deletion

**Figure 2:**
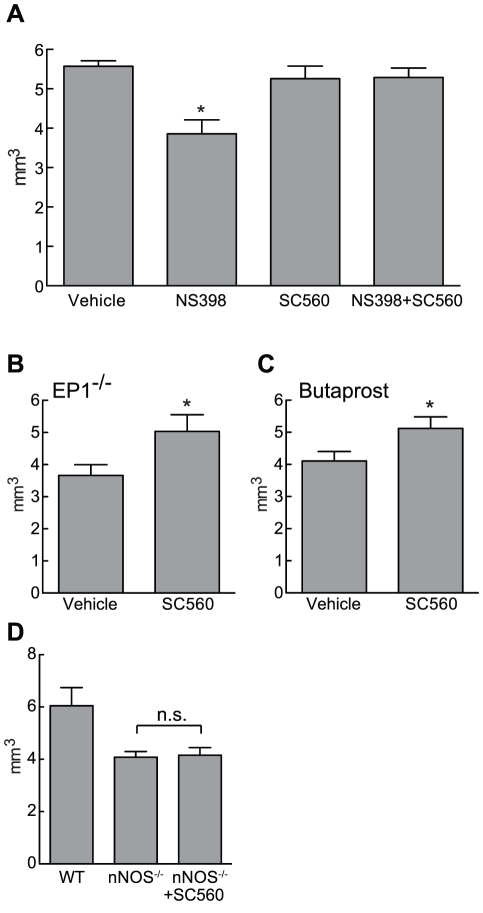
COX-1 inhibition reverses protection obtained by COX-2 inhibition, EP1 receptor deletion, and EP2 receptor activation. (**A**) Cortical lesion volumes were determined from thionine stained serial brain sections 24 hours after NMDA injection. Where indicated, animals received NS398 and/or SC560 as described in *Methods*. Vehicle consisted of 100 µl DMSO∶Saline (1∶9) administered i.p. (n = 6–9/group, * p<0.05). (**B**) NMDA lesion volumes in EP1^−/−^ mice systemically treated with vehicle (as in A) or SC560 (n = 5/group, * p<0.05 from vehicle). (**C**) Mice received the EP2 receptor agonist Butaprost (170 fmol) together with NMDA resulting in reduced lesion volumes. Lesions are enlarged in mice receiving SC560 (vehicle as in A; n = 9/group; * p<0.05 from vehicle). (**D**) Reduced lesion in nNOS^−/−^ mice is not reversed by SC560 (n = 6/group; n.s. = non significant).

Results from our laboratory have provided evidence that COX-2, but not COX-1, is involved in the cortical lesions produced by NMDA [11], [17]. We have also shown that PGE_2_, and not other COX-2 reaction products mediate the neurotoxic effect [16]. Because COX-1 activity contributes substantially to NMDA-induced PGE_2_ production (Fig. 1A), we addressed the relative contribution of COX-1 and COX-2 to the development of excitotoxic lesions by pharmacologic inhibition of one or both enzymes. Consistent with previous findings, the lesion produced by NMDA was attenuated in mice treated with NS398, but not SC560 (Fig. 2A). However, treatment with both inhibitors abolished the protective effect obtained with COX-2 inhibition alone (Fig. 2A). EP1 receptors are the downstream effector of COX-2 mediated neurotoxicity [6], [7]. Therefore, we used EP1^−/−^ mice to investigate the role of COX-1 in the protective effect of EP1 deletion. As previously reported, lesion size was reduced in EP1^−/−^ mice (−33±5% compared to wild type animals, Fig. 2B). However, when EP1^−/−^ mice were treated with SC560 the lesion volume increased and was comparable to that observed in wild type mice (Fig. 2B). Because PGE_2_ can have neuroprotective effects through activation of EP2 receptors [8], we also investigated the role of COX-1 in the neuroprotection induced by EP2 receptor activation. The EP2 receptor agonist butaprost reduced NMDA-induced lesions (−27±4% compared to vehicle control), but the protection was abolished in mice treated with SC560 (Fig. 2C). However, SC560 failed to reverse the protection observed in nNOS−/− mice (Fig. 2D). Therefore, COX-1 activity is required for the protective effect exerted by COX-2 inhibition, EP1 receptor deletion and EP2 receptor activation, but might not play a role in other excitotoxic pathways.

### COX-1 and COX-2 are expressed in different cell types in the mouse cortex

**Figure 3:**
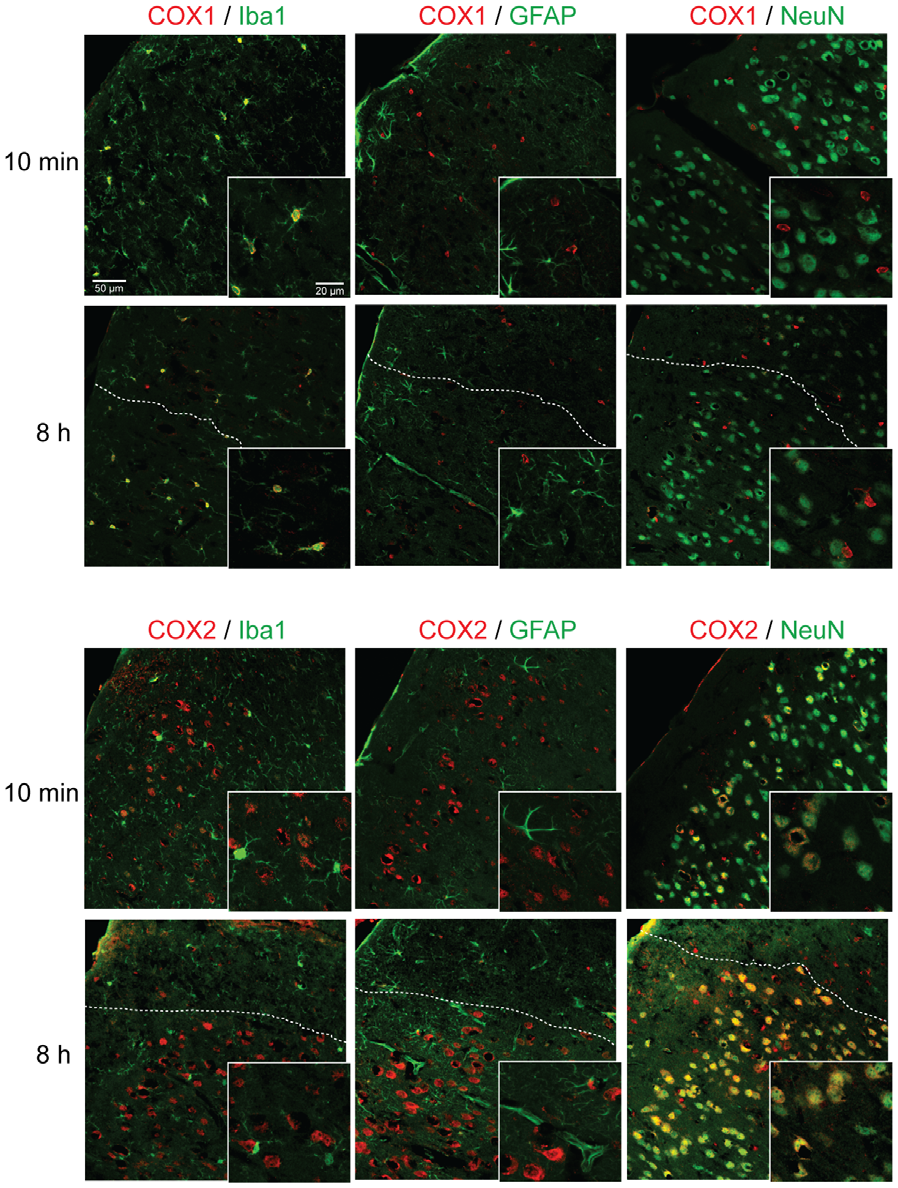
COX-1 and COX-2 expression in the mouse cerebral cortex. Double immunofluorescence labeling of COX enzymes together with Iba1 (microglia), GFAP (astrocytes), or NeuN (neurons) marker proteins 10 minutes or 8 hours after cortical NMDA injections. COX-1 IR co-localizes with Iba1, but not with NeuN or GFAP. COX-2 IR co-localizes with NeuN, but not with GFAP. There is some discrete COX-2 IR associated with Iba1 positive cells. The dotted line marks the lesion border in 8 hour samples. Lesion areas also show reduced NeuN and COX-2 IR.

The observation of a distinct temporal profile of PGE_2_ synthesis by COX-1 and COX-2, let us to ask whether expression of the two COX isoforms occurs in different cell types. Therefore, we examined the cellular localization of COX-1 and COX-2 immunoreactivity (IR) in the mouse cortex by double labeling with neuronal, astrocytic, and microglial markers. At 10 minutes after cortical NMDA injection, COX-2 IR was localized primarily in neurons, exhibiting the well-described perinuclear staining pattern [26] (Fig. 3). Because the neuronal marker NeuN is only expressed in the nucleus and perikaryon we could not examine localization of COX-2 in axons or dendrites. Consistent with previous findings [27], not all neurons showed detectable COX-2 IR. Occasionally, COX-2 IR could be seen in microglia, in which a condensed perinuclear staining pattern was observed (Fig. 3). COX-2 IR was not observed in GFAP-positive astrocytes (Fig. 3). At 8 hours after NMDA, COX-2 IR was markedly increased in neurons of the perilesional cortex. However, COX-2 IR was lost in the lesioned area, together with NeuN IR, most likely reflecting neuronal cell loss. As previously reported [28], [29] COX-1 IR was primarily observed in Iba1 positive microglia, wherein it showed a diffuse cytoplasmic staining pattern. Most identified microglia showed COX-1 IR. Eight hours after NMDA injection COX-1 IR was still confined to Iba1 positive cells (Fig. 3).

### ATP stimulates COX-1 dependent PGE_2_ production in microglia *in vitro*

**Figure 4:**
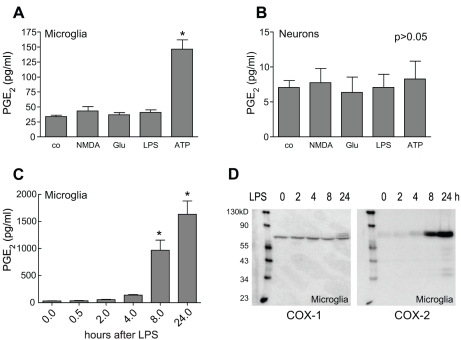
ATP is an immediate inducer of microglial PGE_2_ production. Cultured cortical microglia (**A**) or neurons (**B**) were exposed to NMDA (1 mM), glutamate (Glu; 300 µM), LPS (1 µg/ml) or ATP (1 mM) for 30 minutes. PGE_2_ levels in supernatants were determined by ELISA (n = 6/group derived from 3 independent experiments, * p<0.05). (**C**) Microglia were exposed to LPS (1 µg/ml) for indicated time points and PGE_2_ was assayed in the supernatant by ELISA (n = 6/group derived from 3 independent experiments). (**D**) Microglial COX-1 and COX-2 expression levels after LPS (1 µg/ml) treatment were determined by western blot analysis. Blots are representative for four independent experiments.

Having established that COX-1 expression is limited to microglia we investigated circumstances that would induce PGE_2_ release from these cells *in vitro*. Cultured mouse primary cortical microglia were treated with NMDA (1 mM), glutamate (300 µM), LPS (1 µg/ml), or ATP (1 mM) for 30 minutes and PGE_2_ levels were assessed in the supernatant. Only ATP induced robust PGE_2_ production (Fig. 4A). In contrast, PGE_2_ production was unchanged in neurons treated with ATP or the other agonists studied (Fig. 4B). LPS has been shown to induce PGE_2_ production in microglia [30], [31], but we failed to detect PGE_2_ 30 minutes after LPS treatment. Therefore, we asked whether LPS could induce PGE_2_ release at later time points. LPS strongly induced PGE_2_ release after 8 and 24 hours exposure (Fig. 4C). This increase was mirrored by up-regulation of COX-2 protein that was barely detectible in non-stimulated microglia, while COX-1 was constitutively expressed and protein levels were not altered by LPS treatment (Fig. 4D).

### P2X7 receptors are essential for ATP induced microglial PGE_2_ production *in vitro*

**Figure 5:**
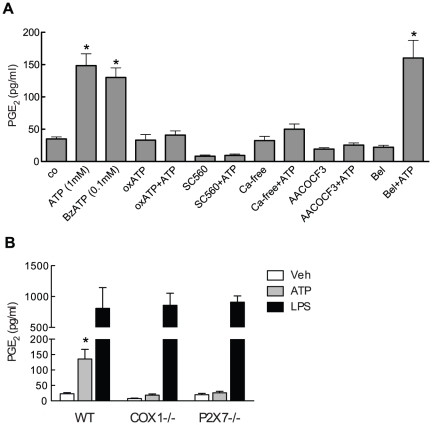
P2X7 receptors mediate ATP but not LPS induced PGE_2_ release in microglial cultures. (**A**) Microglia were treated with ATP (1 mM) or the P2X7 receptor agonist BzATP (100 µM) alone or together with the P2X7 receptor inhibitor oxATP (1 mM), the PLA2 inhibitor AACOCF3 (20 µM), the COX-1 inhibitor SC560 (1 µM), or the Ca^2+^-independent PLA2 inhibitor BEL (10 µM). Some experiments were conducted in Ca^2+^-free extracellular solution (Ca-free). PGE_2_ levels in supernatants were determined by ELISA (n = 8–12/group derived from 4–6 independent experiments, * p<0.05 from all others except ATP, BzATP, and BEL+ATP groups). (**B**) Microglia derived from wild type, COX1^−/−^, or P2X7^−/−^ animals were stimulated with ATP (1 mM) for 30 minutes or LPS (1 µg/ml) for 16 hours. Vehicle consisted of 5 µl PBS. PGE_2_ levels in supernatants were determined by ELISA (n = 6/group derived from 3 independent experiments, * p<0.05 from other groups with same treatment).

Next we examined the signaling cascade that leads to ATP-dependent PGE_2_ production in microglia. The fact that PGE_2_ production was observed when cells were stimulated with high (1 mM), but not low (10–100 µM; data not shown), concentrations of ATP suggested that microglial purinergic receptors of the P2X7 subtype may be involved [32]. Consistent with this hypothesis the preferential P2X7 receptor agonist BzATP was able to induce the same level of PGE_2_ production at a 10-fold lower concentration than ATP while the irreversible P2X7 receptor antagonist oxATP completely blocked ATP induced PGE_2_ release (Fig. 5A). SC560 blocked ATP-induced PGE_2_ production, attesting to the involvement of COX-1 in the process. The Ca^2+^-dependent enzyme phospholipase A2 (PLA2) releases arachidonic acid from cell membrane phospholipids, which, in turn, serves as substrate for COX-dependent PGH_2_ synthesis [33]. Because P2X7 receptors are ATP gated ion channels, we examined whether Ca^2+^ influx and PLA2 activation are required for the COX-1-dependent PGE_2_ production induced by ATP. We found that PGE_2_ production is virtually abolished by removal of extracellular Ca^2+^ (Fig. 5A). Furthermore, the non-selective PLA2 inhibitor AACOCF3 blocked PGE_2_ production, while the Ca^2+^-independent PLA2 inhibitor BEL had no effect (Fig. 5A). Accordingly, ATP induced PGE_2_ release was abolished in microglia derived from COX-1^−/−^ or P2X7^−/−^ animals, while PGE_2_ release after 16 hours LPS stimulation, which is mainly COX-2 dependent (Fig. 4C and 4D), was not affected (Fig. 5B). Collectively these observations indicate that ATP induces PGE_2_ production through Ca^2+^ entry via P2X7 receptors and subsequent cPLA2 and COX-1 activation.

### NMDA elicits ATP release from cultured cortical neurons and induces PGE_2_ production in neuron-microglia co-cultures in a P2X7 receptor dependent manner

**Figure 6:**
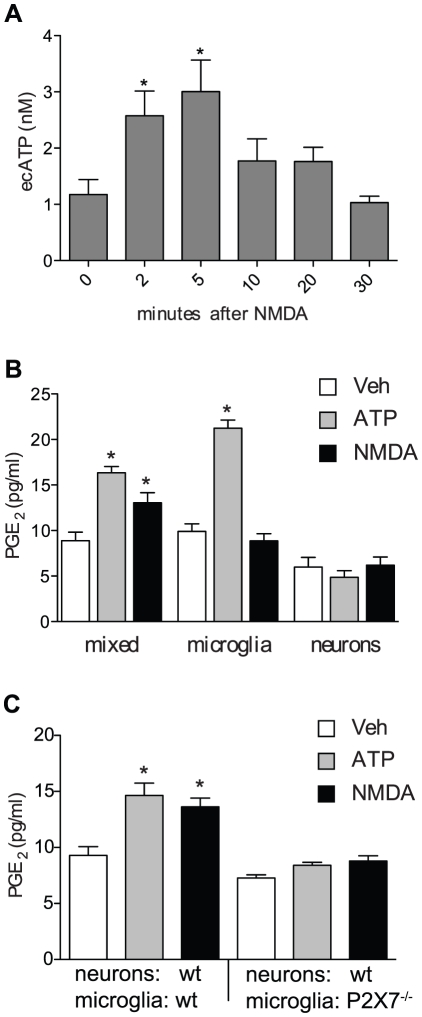
NMDA triggers neuronal ATP release and induces PGE_2_ production in neuron-microglia co-cultures. (**A**) Neurons were exposed to NMDA (1 mM) and extracellular ATP (ecATP) levels were determined in the supernatant (n = 8/group derived from 4 independent experiments, * p<0.05 from 0). (**B**) Co-cultured microglia/neurons or separately cultured microglial and neuronal sister cultures were exposed to ATP (1 mM) or NMDA (1 mM) for 30 minutes. Vehicle consisted of 5 µl PBS. PGE_2_ was determined in supernatants (n = 12/group derived from 6 independent experiments, * p<0.05 from vehicle of the respective group). (**C**) wt neurons were co-cultured with wt or P2X7^−/−^ microglia and stimulated as described in B (n = 6/group derived from 3 independent experiments, * p<0.05 from vehicle of the respective group).

Our *in vivo* findings indicate that the COX-1-dependent early PGE_2_ production is mediated by NMDA receptor activation (Fig. 1B), while our *in vitro* studies revealed a central role for microglial P2X7 receptors in COX-1-dependent PGE_2_ production after ATP exposure. Therefore, we hypothesized that neurons might release extracellular ATP after NMDA receptor activation that in turn would activate P2X7 receptors on microglia resulting in COX-1 activation and PG production. To test this hypothesis we treated cortical neuron cultures with NMDA (1 mM) and determined ATP in the culture medium at different time points after NMDA addition. NMDA triggered ATP release at 2 and 5 minutes (Fig. 6A). No significant ATP increase was observed at later time points.

Asserting the role of neuronal ATP in driving microglial PG production, we found that NMDA induced PGE_2_ production in neuron-microglia co-cultures, but not in pure neuronal or microglial sister cultures (Fig. 6B). When wild type neurons were co-cultured with P2X7^−/−^ microglia no PGE_2_ production was observed after NMDA or ATP treatment (Fig. 6C). These findings support the conclusion that neuronal ATP release after NMDA stimulation triggers microglial PGE_2_ production through P2X7 receptor activation.

### P2X7 receptors contribute to NMDA induced PGE_2_ production *in vivo* and have an essential role in the neuroprotection conferred by the COX-2 inhibition

**Figure 7:**
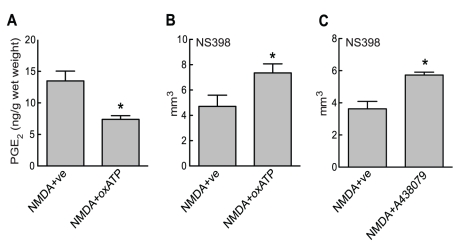
*In vivo* inhibition of P2X7 receptors blocks NMDA induced PGE_2_ production and reverts the protective effect of NS398. (**A**) Animals were injected i.c.v. with saline (ve; 3 µl) or the P2X7 receptor blocker oxATP (300 nmol in 3 µl). Thirty minutes later NMDA was delivered stereotaxically to the parietal cortex. Brain tissue was collected 10 minutes after end of NMDA injection and PGE_2_ tissue levels were determined (n = 5/group; * p<0.05 from NMDA+ve). (**B**) Animals were treated as in A and both groups received NS398 (20 mg/kg i.p.). Lesion volumes were determined 24 hours after NMDA injections (n = 5/group; * p<0.05 from NMDA+ve). Note the slightly increased lesion volumes in NS398+ve treated as compared to animals presented in Fig. 2A and 7C, a fact that could be related to surgical procedures associated with i.c.v. injections. (**C**) Animals received NS398 (20 mg/kg i.p.) together with A438079 (200 µmol/kg in physiol. saline; i.p.) or vehicle (100 µl physiol. saline; i.p.). Lesion volumes were determined 24 hours after NMDA injections (n = 4/group; * p<0.05 from NMDA+ve).

Having established that P2X7 receptors are upstream of COX-1 mediated PGE_2_ production in microglia and that ATP released from neurons after NMDA receptor activation might be a trigger for their activation, we tested the hypothesis that P2X7 receptors also mediate COX-1 dependent PGE_2_ production after NMDA challenge *in vivo*. Because early PGE_2_ release in this excitotoxic model is solely COX-1 dependent (Fig. 1), we examined PGE_2_ production 10 minutes after NMDA injection in mice that have been pretreated with the P2X7 receptor blocker oxATP. PGE_2_ levels were significantly decreased in animals pretreated with oxATP when compared to animals receiving vehicle alone (Fig. 7A). Furthermore, similar to the effects seen after COX-1 inhibition, pre-treatment with oxATP or with the selective P2X7 receptor antagonist A438079 reversed the protection offered by NS398 treatment, confirming that P2X7 receptors are essential for COX-1 mediated prostanoid production after cortical NMDA injections (Fig. 7B and 7C).

## Discussion

Purinergic receptors play a central role in shaping the microglial response to physiologic and pathologic environmental cues [34]. Here we identified P2X7 receptors as regulators of COX-1 dependent PGE_2_ production in microglia and determined the contribution of this signaling pathway to the development of excitotoxic cortical brain lesions.

We show that ATP is a strong inducer of COX-1 dependent PGE_2_ production in primary microglial cultures. The fact that microglial PGE_2_ release occurred within 30 minutes after ATP exposure makes it likely that the release is dependent on the activation of a constitutive synthetic machinery, rather than on transcriptional induction of such components. COX-1 is the predominant cyclooxygenase isoform expressed in resting microglia *in vivo* (Fig. 3) and *in vitro* (Fig. 4D). In contrast little constitutive COX-2 protein is expressed and up-regulation requires prolonged exposure to a pro-inflammatory stimulus such as LPS (Fig. 4D). Microglia express several purinergic receptors, including members of the G-protein coupled P2Y family and ionotropic P2X receptors [34]. Here we present evidence that P2X7 receptors are instrumental for ATP induced PGE_2_ release in microglia. First, relatively high (mM) ATP concentrations are needed to elicit PGE_2_ production. Low potency of ATP is a signature of the P2X7 receptor, while P2Y and other P2X receptors are activated at 10 fold lower concentration [35]. Second, exposure of microglia to 100 µM BzATP is as effective as 1 mM ATP in releasing PGE_2_. Although capable of binding other P2X receptors, BzATP shows high potency only for the P2X1 and P2X7 receptor [36]. Third, oxATP, which is an irreversible inhibitor with good specificity for the P2X7 receptor [37], efficiently blocked microglial PGE_2_ production. Finally, the ATP response was blunted in P2X7^−/−^ microglia.

Our data suggest that cPLA2 is activated in response to P2X7 receptor activation. Thus, PGE_2_ production in our model is dependent on extracellular Ca^2+^ and is inhibited by the non-selective PLA2 blocker AACOCF3. The observation that BEL, an inhibitor of Ca^2+^-independent cPLA2γ, does not attenuate PGE_2_ production indicates the involvement of Ca^2+^ dependent cPLA2α activity.

We also demonstrated that NMDA receptor activation results in rapid ATP release from cortical neurons. ATP may be secreted by neuronal activity from pre-synaptic vesicles where ATP concentrations can be as high as 150 mM [38]. Indeed, glutamate-induced ATP release has been reported in Xenopus spinal neurons [39]. Alternatively, ATP can be released from neurons as a result of injury when cytoplasmic ATP, which is estimated to be in the range of 10–100 mM, leaks to the extracellular space. Consistent with this scenario, focal cerebral ischemia in rats results in early increases in extracellular ATP [40]. It is therefore conceivable that mM concentrations of ATP may be reached in the vicinity of microglia after exposure to excitotoxic levels of NMDA, resulting in P2X7 receptor activation. This interpretation is supported by our findings that NMDA elicits PGE_2_ production in neuron-microglia co-cultures, but not in pure neuronal or microglial cultures, and that this production is dependent on microglial P2X7 receptors.

We assessed the relative contribution of COX-1 and COX-2 in NMDA induced PGE_2_ production and tissue damage in the mouse cortex. As previously reported [11], [17], pharmacological inhibition of COX-2 reduced lesion volumes, while COX-1 inhibition had no effect. However, inhibition of both isoforms abolished the protection observed with COX-2 inhibition. Thus, COX-1-derived reaction products do not reduce NMDA-induced brain damage, but are able to protect the brain if COX-2 is inhibited. Similarly, COX-1 inhibition abolished the protection observed in mice lacking EP1 receptors, and the protective effect induced by EP2 receptor activation. COX-1 was observed exclusively in microglia, and was activated by purinergic signaling leading to cPLA2 activation. Experiments in neuron-microglia co-cultures indicated that neurons are the source of ATP driving purinergic signaling and COX-1 activity. The data provide new evidence for a neuroprotective role of microglial COX-1 in the setting of excitotoxic brain injury.

The cortical PGE_2_ production induced by excitotoxicity follows a biphasic temporal profile. While COX-1 accounts for the early PGE_2_ release, which peaks 10 minutes after NMDA injection, both COX-1 and COX-2 contribute to the late PGE_2_ production that is maximal at 8 hours after the NMDA challenge. Co-administration of COX-1 and COX-2 inhibitors lowered PGE_2_ below baseline levels for at least 24 hrs, indicating that the concentrations used were sufficient to block both enzymes throughout the experiment. Interestingly, the early PGE_2_ production was only partially dependent on NMDA receptor activation, because MK-801 did not reduce PGE_2_ down to baseline levels. Indeed, vehicle injection was sufficient to trigger PGE_2_ release, which, in contrast to NMDA, could not be blocked by MK-801. It is therefore likely that the NMDA-independent component of the early PGE_2_ production is related to the trauma caused by insertion of the micropipette. Similar cortical injuries have been shown to cause purine release from damaged cells [41], and it is tempting to speculate that these purines activate COX-1-dependent PGE_2_ production through microglial purinergic receptors.

The mechanisms of the protective effect exerted by COX-1 in the setting of COX-2 inhibition have not been elucidated. One possibility is that COX-1-released PGE_2_ preferentially acts on cytoprotective G_s_-coupled EP receptors, such as EP2 or EP4 [8], [42], [43]. Our data do not rule out this possibility, although the reversal of EP2 receptor mediated neuroprotection by COX-1 inhibition would suggest that the EP2 receptor is not the target for COX-1 derived PGE_2_. The role of EP4 receptors in this paradigm remains to be explored. Another possibility is that, in addition to PGE_2_, other prostanoids released by microglia play a role in the protection. Thus, microglial COX-1 could be linked to PG synthases distinct from those associated with neuronal COX-2, resulting in synthesis of different prostanoids in the two cell types. It has become clear that several prostanoid receptors can initiate neuroprotective signaling [44]. In this respect it is interesting that mice deficient in the hematopoietic PGD_2_ synthase isoform, which in brain is exclusively expressed in microglia and perivascular macrophages, are more susceptible to hypoxic-ischemic damage [45]. This effect is coupled to activation of G-coupled DP1 receptors which are neuroprotective [45], [46].

Alternatively, COX-2 and COX-1 derived prostanoids might have different cellular targets. We have previously established that EP1 receptors mediate COX-2 neurotoxicity [7]. Because COX-1 inhibition reverts the protected phenotype in EP1^−/−^ mice, it could be argued that COX-1-derived prostanoids counteract a pathogenic mechanism that is not directly linked to neuronal COX-2. This assumption is also supported by the fact that EP2 receptor activation, which is directly neuroprotective by rising intracellular cAMP levels [8], could not override the deleterious effect of COX-1 inhibition (Fig. 2). Although in the excitotoxic model used in the present study neuronal cell fate is mainly dependent on intrinsic neuronal mechanisms, i.e. energy depletion, intracellular Ca^2+^ overload, oxidative stress, protease activation, and mitochondrial dysfunction [47], involvement of microglia has also been suggested [48], [49]. Because PGD_2_ is the precursor for the peroxisome proliferators activator receptor-γ (PPARγ) ligand 15-Deoxy-^Δ12,14^-PGJ_2_, it is possible that COX-1 might be linked to PPARγ activation which has been shown to mitigate microglial pro-inflammatory response in a variety of models [50]. Thus COX-1 might be part of an autocrine loop to limit microglia activation after excitotoxic brain lesions. However, the protective effect of COX-1 derived prostanoids might be limited to excitotoxic neuronal cell death. For example, in contrast to our findings, COX-1 contributed to neuronal cell death after neuroinflammation triggered by intracerebroventricular LPS injection [51]. Interestingly, COX-1 inhibition did not reverse the protected phenotype of nNOS^−/−^ animals. Thus, the neuroprotective contribution of COX-1 is likely to be pathway specific, and might be confined to the cerebral prostanoid system.

In addition, we cannot exclude the involvement of lipid mediators not directly produced by the COX system. Apart from COX, arachidonic acid can be metabolized by lipoxygenases to form leukotrienes and lipoxins and the cytochrome P450 pathway to form epoxyeicosatrienoic acids (EETs) and hydroxyeicosatetraenoic acids (HETEs) [52]. Blocking COX could therefore shunt arachidonic acid released after P2X7 receptor activation to be utilized by these pathways. Evidence suggests that several lipoxygenase and P450 epoxygenase metabolites are neurotoxic [53]. It is therefore conceivable that COX-1 inhibition could increase lipoxygenase or cytochrome P450-derived neurotoxic lipid mediators leading to a reversal of the neuroprotection granted by COX-2 inhibition. It is however not clear why these neurotoxins would be only relevant when COX-2 is inhibited and not be neurotoxic in naïve animals or in nNOS^−/−^ mice.

Finally we show that P2X7 receptors are pivotal for COX-1 activation after cortical NMDA injections *in vivo*. Animals pretreated with the P2X7 receptor blocker oxATP showed reduced early PGE_2_ production after cortical NMDA injection, indicating that P2X7 receptors are activators of COX-1 mediated PGE_2_ production in the setting of excitotoxic brain lesions. As expected, we found that P2X7 receptor inhibition by oxATP or the competitive antagonist A438079, similar to COX-1 inhibition, reversed the protective effect afforded by the COX-2 inhibitor NS398. This data further confirm that NMDA induced COX-1 activity is dependent on purinergic signaling through the P2X7 receptor.

In summary, we have provided evidence that COX-1 is solely responsible for the early increase in PGE_2_ induced by cortical injection of NMDA, and that COX-1 activity contributes to the protective effect exerted by COX-2 inhibition, EP1 receptor deletion, or EP2 receptor activation. In the cortex of naïve animals, COX-1 is localized to microglia, whereas COX-2 is present mainly in neurons. Microglial COX-1 coupled to PGE_2_ synthases is able to synthesize PGE_2_ in response to extracellular ATP, probably released by an effect of NMDA on neurons rather than microglia. COX-1 catalytic activity is increased as a result of cPLA2α activation following Ca^2+^ influx through the P2X7 receptor. These findings, collectively, suggest that neuronal release of ATP following NMDA stimulation leads to activation of COX-1 in microglia. Although the mechanisms by which COX-1-derived prostanoids influence lesion outcome remain to be defined, the data indicate that COX-1 in microglia is endowed with a powerful neuroprotective potential, which could be beneficial in the treatment of neurological diseases associated with excitotoxicity.
